# Development and validation of a prediction model for hypotension after neuraxial anesthesia in preeclamptic parturients: a multicenter retrospective study

**DOI:** 10.3389/fmed.2025.1649657

**Published:** 2025-10-13

**Authors:** Jie Su, Qumin Chen, Xiuhong Ye, Huan Lu

**Affiliations:** ^1^Department of Anesthesiology, Fuzhou Jin’an District Hospital, Fuzhou, Fujian, China; ^2^Department of Anesthesiology, The First Affiliated Hospital of Xiamen University, School of Medicine, Xiamen University, Xiamen, China; ^3^Department of Anesthesiology, Zhangzhou Affiliated Hospital of Fujian Medical University, Zhangzhou, Fujian, China; ^4^Department of Anesthesiology, Fujian Maternity and Child Health Hospital, Fuzhou, Fujian, China

**Keywords:** preeclampsia, neuraxial anesthesia, hypotension prediction, nomogram validation, obstetric anesthesia

## Abstract

**Objective:**

To develop and validate a multivariate prediction model for hypotension following neuraxial anesthesia in preeclamptic parturients.

**Methods:**

This multicenter retrospective study analyzed 1,402 preeclamptic parturients (gestational age ≥28 weeks) from three tertiary centers (2013–2024). After exclusions (*n* = 569), 833 patients were allocated to training (*n* = 495), internal validation (*n* = 213), and external validation (*n* = 125) cohorts. Multivariable logistic regression identified independent predictors, with subsequent nomogram construction. Model performance was assessed via discrimination (AUC), calibration (Hosmer-Lemeshow), and clinical utility [decision curve analysis (DCA), clinical impact curves (CIC)].

**Results:**

Seven independent predictors were identified: platelet count (OR 0.920, 95%CI 0.876–0.966), sFlt-1/PlGF ratio (OR 1.039, 95%CI 1.002–1.078), baseline perfusion index (OR 0.221, 95%CI 0.101–0.485), T6 anesthesia level (OR 11.353, 95%CI 1.408–29.320), local anesthetic dose (OR 29.391, 95%CI 4.792–38.270), fetal weight (OR 1.004, *p* = 0.045), and umbilical artery S/D ratio (OR 9.319, *p* < 0.001). The nomogram demonstrated robust discrimination (training AUC 0.851; internal validation AUC 0.836; external validation AUC 0.810) and calibration (mean absolute errors: 0.013–0.038). DCA confirmed clinical utility at a 45% risk threshold (net benefit 0.62), capturing 85% of events with 32% false positives.

**Conclusion:**

This validated prediction model accurately stratifies hypotension risk in preeclamptic parturients receiving neuraxial anesthesia. The nomogram facilitates targeted prophylactic interventions, optimizing resource allocation and maternal hemodynamic stability.

## 1 Introduction

Neuraxial anesthesia remains the preferred technique for cesarean delivery in preeclamptic parturients due to its hemodynamic stability and reduced risk of airway complications compared to general anesthesia (1, 2). However, post-neuraxial hypotension—occurring in 30–60% of this population—poses significant maternal and fetal hazards, including uteroplacental hypoperfusion, fetal acidosis, and emergent interventions (3–5). Preeclampsia’s unique pathophysiology, characterized by endothelial dysfunction, angiogenic imbalance (elevated sFlt-1/PlGF ratio), and intravascular volume contraction, amplifies this risk (6–9). Current reactive strategies, such as vasopressor administration for established hypotension, address consequences rather than prevention and may fail to mitigate initial hemodynamic insults. Consequently, proactive risk stratification is essential to guide timely prophylactic measures.

Existing prediction tools exhibit critical limitations: they often derive from homogeneous cohorts excluding preeclampsia, overlook disease-specific biomarkers (e.g., angiogenic factors and perfusion indices), and lack multicenter validation (2, 3, 5). While recent efforts focus on comparing vasopressor efficacy (e.g., norepinephrine’s reduced bradycardia risk versus phenylephrine), no validated model integrates preeclampsia’s multifactorial hemodynamic determinants to individualize prophylaxis (2–5, 10). This gap impedes targeted resource allocation and perpetuates variability in clinical outcomes.

To address these limitations, we developed and validated a multivariate prediction model for post-neuraxial hypotension in preeclamptic parturients. Our model uniquely incorporates seven pathophysiologically grounded predictors—platelet count, sFlt-1/PlGF ratio, baseline perfusion index, sensory blockade level, local anesthetic dose, fetal weight, and umbilical artery S/D ratio—reflecting preeclampsia’s hemodynamic, autonomic, and fetoplacental dimensions. Leveraging a multicenter retrospective design with rigorous external validation, this study aims to provide a clinically deployable tool for precision anesthesia management in this high-risk population.

## 2 Materials and methods

### 2.1 Data source

**Figure 1:**
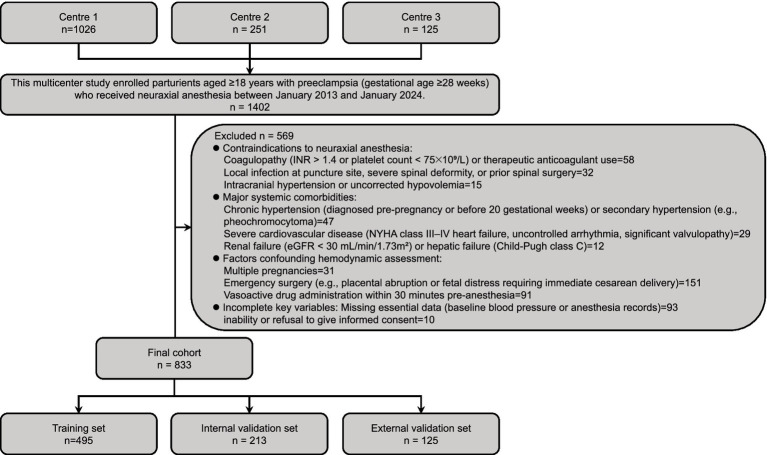
Patient enrollment flowchart and cohort stratification. Schematic representation of multicenter participant selection, exclusion criteria application, and cohort allocation. Initial screening identified 1,402 preeclamptic parturients (gestational age ≥28 weeks) receiving neuraxial anesthesia (January 2013–January 2024) across three centers. Following application of rigorous exclusion criteria (*n* = 569)—including contraindications to neuraxial anesthesia (*n* = 105), major systemic comorbidities (*n* = 88), hemodynamic assessment confounders (*n* = 273), and incomplete data (*n* = 103)—833 eligible patients comprised the final cohort. These were randomly allocated to training (*n* = 495) and internal validation (*n* = 213) sets (7:3 ratio). An independent external validation cohort (*n* = 125) from Center 3 was incorporated to assess model generalizability.

This multicenter retrospective cohort study utilized clinical data extracted from tertiary hospitals specializing in obstetric care between January 2013 and January 2024. Electronic health records, anesthesia management systems, and institutional databases were systematically reviewed. Patient demographics, clinical characteristics, and detailed anesthesia records underwent blinded verification by two independent reviewers. From an initial pool of 1,402 eligible parturients meeting inclusion criteria (diagnosis of preeclampsia per ACOG/ISSHP criteria; gestational age ≥28 weeks; maternal age ≥18 years; receipt of neuraxial anesthesia [epidural, spinal, or combined spinal-epidural] with complete hemodynamic documentation), exclusion criteria were sequentially applied: contraindications to neuraxial anesthesia (coagulopathy [INR > 1.4 or platelets <75 × 10^9^/L]), therapeutic anticoagulation, spinal deformity, local infection, uncorrected hypovolemia, intracranial hypertension; significant comorbidities (chronic hypertension, secondary hypertension, NYHA class III/IV heart failure, uncontrolled arrhythmias, severe valvular disease, renal failure [eGFR <30 mL/min/1.73m^2^], or hepatic failure [Child-Pugh C]); multiple pregnancies; emergency surgery (e.g., placental abruption); vasoactive medication administration within 30 min pre-anesthesia; or missing critical data (e.g., baseline blood pressure, anesthetic agents; Inability or refusal to give informed consent). This resulted in a final analytical cohort of 833 patients (Figure 1). Standardized protocols ensured comprehensive data collection on maternal status, anesthetic management, and hemodynamic outcomes.

### 2.2 Study definitions

Hypotension was defined as a reduction in systolic blood pressure (SBP) exceeding 30% from baseline or an absolute SBP < 90 mmHg within 30 min after neuraxial anesthesia administration. Severe hypotension was specified as SBP < 80 mmHg persisting for >3 min. Baseline SBP was calculated as the mean of three consecutive measurements obtained ≤10 min prior to anesthesia induction (2, 11, 12). (1) Demographic and baseline characteristics included: Pre-pregnancy BMI: Body mass index (kg/m^2^) calculated using self-reported pre-gestational weight and height; Preeclampsia type: Classified as early-onset (diagnosis <34 weeks’ gestation) or late-onset (≥34 weeks) per ISSHP-ACOG criteria (13); Preeclampsia severity: Mild (SBP 140–159 mmHg and/or DBP 90–109 mmHg without end-organ dysfunction) or severe (SBP ≥ 160 mmHg, DBP ≥ 110 mmHg, or evidence of hepatic/renal/neurological involvement); Platelet count and hepatic transaminases (ALT/AST): Most recent values recorded within 24 h preceding anesthesia. (2) Placental function biomarkers (measured ≤24 h pre-anesthesia): sFlt-1/PlGF ratio: Soluble fms-like tyrosine kinase-1 to placental growth factor ratio, quantified via electrochemiluminescence immunoassay; Urine protein: 24-h urinary protein excretion (g/24 h); PAPP-A: Pregnancy-associated plasma protein-A (mIU/mL), assessed using chemiluminescence. (3) Hemodynamic parameters: Baseline SBP/DBP: Mean of three measurements ≤10 min pre-anesthesia; Perfusion index (PI): Pulse oximetry-derived ratio of pulsatile to non-pulsatile blood flow (%), recorded after 5 min of supine rest pre-induction; Blood pressure variability (BPV): Standard deviation of SBP divided by mean SBP × 100%, calculated from continuous non-invasive monitoring during the 10-min period preceding anesthesia; Heart rate variability (HRV): Root mean square of successive differences (RMSSD, ms) in R-R intervals from 5-min electrocardiography pre-induction. (4) Anesthesia-related factors: Sensory block level: Highest dermatomal blockade assessed by loss of cold sensation (ice test) 10 min post-injection; Local anesthetic dose: Total intrathecal ropivacaine equivalent (mg), adjusted for potency relative to bupivacaine; Preload fluid: Crystalloid volume (mL) administered intravenously 30 min pre-anesthesia; Time to incision: Interval (minutes) from local anesthetic injection to surgical incision. (5) Fetal and obstetric parameters: Estimated fetal weight: Predicted weight (g) via ultrasound (Hadlock formula) ≤ 24 h pre-procedure; Umbilical artery S/D ratio: Systolic-to-diastolic velocity ratio from Doppler ultrasonography ≤24 h pre-delivery (13); Non-stress test (NST): Reactive (≥2 accelerations in 20 min) or non-reactive per ACOG guidelines (13). (6) Outcome measures: Vasopressor total dose: Cumulative intraoperative norepinephrine-equivalent dose (μg), with phenylephrine converted at 12.5:1 potency ratio; Fetal acidosis: Umbilical artery pH < 7.20 measured ≤5 min post-delivery; SBP nadir: Lowest recorded SBP (mmHg) within 30 min post-anesthesia; All data were collected by trained personnel using standardized perioperative protocols.

### 2.3 Statistical analysis

The study cohort comprised 1,277 parturients from Center 1 (Fujian Maternity and Child Health Hospital, *n* = 1,026) and Center 2 (the First Affiliated Hospital of Xiamen University, *n* = 251). After screening exclusions, 708 eligible cases were randomly allocated to training (70%) and internal validation (30%) sets. To enhance generalizability, an external validation cohort of 125 cases from Center 3 (Fuzhou Jin’an District Hospital) was included. Comparative analyses between hypotension and non-hypotension groups utilized Fisher’s exact or χ^2^ tests for categorical variables and Wilcoxon rank-sum tests for nonparametric continuous data. Significant predictors identified through multivariable logistic regression informed development of a post-anesthesia hypotension nomogram. Model discrimination was evaluated using receiver operating characteristic (ROC) curves, which quantify the ability to distinguish between patients with and without hypotension. Calibration was assessed via Hosmer-Lemeshow tests. Clinical utility was quantified through decision curve analysis (DCA), which evaluates the net benefit of model-guided interventions across probability thresholds, and clinical impact curves (CIC), which illustrate the number of high-risk individuals and true positives identified. Continuous variables are expressed as mean ± standard deviation; categorical variables as counts (percentages). Statistical significance was defined as two-tailed *p* < 0.05. Analyses were performed using R version 4.4.0 and SPSS 26.0.

## 3 Results

### 3.1 Study population and comparative group analysis

The enrollment flowchart (Figure 1) details the screening of 1,402 preeclamptic parturients receiving neuraxial anesthesia across three centers (January 2013–January 2024). Following exclusions (*n* = 569), 833 eligible patients were analyzed, with post-anesthesia hypotension occurring in 48.7% (*n* = 241), 49.3% (*n* = 105), and 47.2% (*n* = 59) of training, internal validation, and external validation cohorts, respectively. Baseline characteristics stratified by hypotension status demonstrated significant intergroup differences across all cohorts (Tables 1–3). Statistically distinct profiles (*p* <0.05) were observed in training cohorts for platelet count, sFlt-1/PlGF ratio, urine protein excretion, baseline diastolic blood pressure, perfusion index, blood pressure variability, local anesthetic dose, anesthesia dermatomal level, estimated fetal weight, umbilical artery systolic/diastolic ratio, and fetal acidosis incidence. Neonatal outcomes consistently exhibited lower umbilical artery pH in hypotensive groups.

**Table 1:** Characteristics of training set.

Subgroup	Non-hypotension group (*n* = 254)	Hypotension group (*n* = 241)	*p*-value*
Age, Mean ± SD	32.05 ± 4.42	31.78 ± 4.71	0.51
Pre BMI, Mean ± SD	28.32 ± 3.87	28.25 ± 4.08	0.85
Parity, *n* (%)			0.02
0	59 (23.23)	56 (23.24)	
1	59 (23.23)	77 (31.95)	
2	53 (20.87)	57 (23.65)	
3	83 (32.68)	51 (21.16)	
PE type, *n* (%)			0.62
Early-onset	95 (37.40)	85 (35.27)	
Late-onset	159 (62.60)	156 (64.73)	
PE severity, *n* (%)			<0.01
Mild	76 (29.92)	99 (41.08)	
Severe	178 (70.08)	142 (58.92)	
Platelet, Mean ± SD	218.09 ± 41.87	164.94 ± 32.78	<0.01
ALT, Mean ± SD	35.37 ± 14.14	35.47 ± 15.36	0.94
AST, Mean ± SD	37.02 ± 14.30	38.22 ± 16.27	0.38
Sflt 1 Plgf Ratio, Mean ± SD	83.56 ± 24.60	127.39 ± 34.35	<0.01
Urine protein, Mean ± SD	2.24 ± 1.10	3.73 ± 1.34	<0.01
PAPP A, Mean ± SD	1.07 ± 0.49	1.07 ± 0.50	0.89
Baseline SBP, Mean ± SD	135.36 ± 11.63	134.17 ± 12.03	0.27
Baseline DBP, Mean ± SD	81.44 ± 5.91	87.63 ± 6.24	<0.01
Baseline PI, Mean ± SD	4.09 ± 0.82	3.06 ± 0.74	<0.01
BPV, Mean ± SD	12.96 ± 3.96	17.54 ± 4.26	<0.01
HRV, Mean ± SD	21.09 ± 5.10	21.14 ± 5.12	0.91
Puncture space, *n* (%)			0.6
L2-3	111 (43.70)	111 (46.06)	
L3-4	143 (56.30)	130 (53.94)	
Anesthesia level, *n* (%)			<0.01
T10	105 (41.34)	58 (24.07)	
T6	76 (29.92)	74 (30.71)	
T8	73 (28.74)	109 (45.23)	
Preload fluid, Mean ± SD	522.59 ± 123.01	521.04 ± 128.13	0.89
LA dose, Mean ± SD	10.31 ± 1.04	12.25 ± 1.07	<0.01
Time to incision, Mean ± SD	10.36 ± 2.52	10.54 ± 2.51	0.41
Gestational weeks, Mean ± SD	35.51 ± 2.35	35.46 ± 2.25	0.8
Fetus weight, Mean ± SD	3056.65 ± 296.78	3225.65 ± 290.95	<0.01
UA SD ratio, Mean ± SD	2.83 ± 0.35	3.57 ± 0.46	<0.01
NST result, *n* (%)			0.31
Non-reactive	42 (16.54)	32 (13.28)	
Reactive	212 (83.46)	209 (86.72)	
SBP min, Mean ± SD	112.30 ± 8.64	76.27 ± 7.95	<0.01
Vasopressor total, Mean ± SD	56.19 ± 31.51	506.91 ± 192.40	<0.01
UA pH, Mean ± SD	7.28 ± 0.05	7.24 ± 0.06	<0.01
Fetal acidosis, *n* (%)			<0.01
No	243 (95.67)	186 (77.18)	
Yes	11 (4.33)	55 (22.82)	

Data are presented as mean ± standard deviation, or number (%). SD, standard deviation; BMI, body mass index; ALT, Alanine Aminotransferase; AST, Aspartate Aminotransferase; BPV, Blood Pressure Variability; DBP, Diastolic Blood Pressure; HRV, Heart Rate Variability; LA, Local Anesthetic; NST, Non-Stress Test; PAPP-A, Pregnancy-Associated Plasma Protein-A; PE, Preeclampsia; PI, Perfusion Index; PlGF, Placental Growth Factor; SBP, Systolic Blood Pressure; SD (in UA SD ratio): Systolic/Diastolic; sFlt-1: soluble fms-like tyrosine kinase-1; UA, Umbilical Artery.

**Table 2:** General characteristics of internal validation set.

Subgroup	Non-hypotension group (*n* = 108)	Hypotension group (*n* = 105)	*p*-value*
Age, Mean ± SD	32.09 ± 6.18	31.58 ± 6.29	0.550
Pre BMI, Mean ± SD	28.57 ± 3.49	28.87 ± 3.62	0.545
Parity, *n* (%)			0.921
0	32 (29.63)	28 (26.67)	
1	29 (26.85)	29 (27.62)	
2	21 (19.44)	19 (18.10)	
3	26 (24.07)	29 (27.62)	
PE type, *n* (%)			<0.001
Early-onset	55 (50.93)	28 (26.67)	
Late-onset	53 (49.07)	77 (73.33)	
PE severity, *n* (%)			<0.001
Mild	60 (55.56)	28 (26.67)	
Severe	48 (44.44)	77 (73.33)	
Platelet, Mean ± SD	224.44 ± 41.22	121.86 ± 22.95	<0.001
ALT, Mean ± SD	73.05 ± 29.28	65.78 ± 31.09	0.081
AST, Mean ± SD	63.69 ± 27.16	63.00 ± 27.83	0.856
Sflt 1 Plgf Ratio, Mean ± SD	51.64 ± 13.37	143.83 ± 33.56	<0.001
Urine protein, Mean ± SD	865.61 ± 326.72	3213.95 ± 1038.32	<0.001
PAPP A, Mean ± SD	1.42 ± 0.66	1.41 ± 0.66	0.954
Baseline SBP, Mean ± SD	135.18 ± 14.26	134.05 ± 14.17	0.563
Baseline DBP, Mean ± SD	87.05 ± 4.61	99.42 ± 6.46	<0.001
Baseline PI, Mean ± SD	4.54 ± 0.85	2.56 ± 0.60	<0.001
BPV, Mean ± SD	10.54 ± 2.59	18.66 ± 3.46	<0.001
HRV, Mean ± SD	50.56 ± 14.74	51.16 ± 14.27	0.764
Puncture space, *n* (%)			0.938
L2-3	56 (51.85)	55 (52.38)	
L3-4	52 (48.15)	50 (47.62)	
Anesthesia level, *n* (%)			<0.001
T10	33 (30.56)	22 (20.95)	
T6	38 (35.19)	66 (62.86)	
T8	37 (34.26)	17 (16.19)	
Preload fluid, Mean ± SD	871.79 ± 68.61	886.07 ± 68.23	0.129
LA dose, Mean ± SD	9.61 ± 0.89	12.45 ± 1.04	<0.001
Time to incision, Mean ± SD	14.66 ± 6.16	15.27 ± 6.14	0.470
Gestational weeks, Mean ± SD	33.00 ± 3.33	33.70 ± 3.45	0.136
Fetus weight, Mean ± SD	2817.92 ± 357.63	3139.77 ± 358.29	<0.001
UA SD ratio, Mean ± SD	2.64 ± 0.34	3.74 ± 0.42	<0.001
NST result, *n* (%)			0.793
Non-reactive	20 (18.52)	18 (17.14)	
Reactive	88 (81.48)	87 (82.86)	
SBP min, Mean ± SD	105.02 ± 8.92	76.12 ± 5.71	<0.001
Vasopressor total, Mean ± SD	86.27 ± 42.12	539.48 ± 170.31	<0.001
UA pH, Mean ± SD	7.30 ± 0.06	7.20 ± 0.06	<0.001
Fetal acidosis, *n* (%)			<0.001
No	108 (100.00)	48 (45.71)	
Yes	0 (0.00)	57 (54.29)	

Data are presented as mean ± standard deviation, or number (%). SD, standard deviation; BMI, body mass index; ALT, Alanine Aminotransferase; AST, Aspartate Aminotransferase; BPV, Blood Pressure Variability; DBP, Diastolic Blood Pressure; HRV, Heart Rate Variability; LA, Local Anesthetic; NST, Non-Stress Test; PAPP-A, Pregnancy-Associated Plasma Protein-A; PE, Preeclampsia; PI, Perfusion Index; PlGF, Placental Growth Factor; SBP, Systolic Blood Pressure; SD (in UA SD ratio): Systolic/Diastolic; sFlt-1, soluble fms-like tyrosine kinase-1; UA, Umbilical Artery.

**Table 3:** General characteristics of external validation set.

Subgroup	Non-hypotension group (*n* = 66)	Hypotension group (*n* = 59)	*p*-value*
Age, Mean ± SD	32.00 ± 6.31	33.12 ± 6.17	0.319
Pre BMI, Mean ± SD	29.19 ± 3.79	28.65 ± 4.05	0.442
Parity, *n* (%)			0.049
0	8 (12.12)	18 (30.51)	
1	19 (28.79)	13 (22.03)	
2	24 (36.36)	13 (22.03)	
3	15 (22.73)	15 (25.42)	
PE type, *n* (%)			0.025
Early-onset	39 (59.09)	23 (38.98)	
Late-onset	27 (40.91)	36 (61.02)	
PE severity, *n* (%)			0.317
Mild	35 (53.03)	26 (44.07)	
Severe	31 (46.97)	33 (55.93)	
Platelet, Mean ± SD	226.76 ± 41.32	115.66 ± 24.47	<0.001
ALT, Mean ± SD	64.44 ± 30.36	67.78 ± 32.78	0.555
AST, Mean ± SD	63.00 ± 26.99	62.69 ± 29.98	0.952
Sflt 1 Plgf Ratio, Mean ± SD	53.85 ± 13.89	147.40 ± 31.74	<0.001
Urine protein, Mean ± SD	874.67 ± 327.27	3364.75 ± 1002.59	<0.001
PAPP A, Mean ± SD	1.35 ± 0.67	1.24 ± 0.60	0.333
Baseline SBP, Mean ± SD	138.38 ± 15.41	134.46 ± 15.01	0.153
Baseline DBP, Mean ± SD	86.82 ± 4.65	98.80 ± 6.20	<0.001
Baseline PI, Mean ± SD	4.44 ± 0.83	2.44 ± 0.61	<0.001
BPV, Mean ± SD	10.38 ± 2.36	17.79 ± 3.82	<0.001
HRV, Mean ± SD	47.33 ± 13.26	53.04 ± 13.52	0.019
Puncture space, *n* (%)			0.342
L2-3	29 (43.94)	21 (35.59)	
L3-4	37 (56.06)	38 (64.41)	
Anesthesia level, *n* (%)			<0.001
T10	18 (27.27)	11 (18.64)	
T6	20 (30.30)	41 (69.49)	
T8	28 (42.42)	7 (11.86)	
Preload fluid, Mean ± SD	705.50 ± 35.51	705.88 ± 28.99	0.947
LA dose, Mean ± SD	9.46 ± 0.79	12.24 ± 0.97	<0.001
Time to incision, Mean ± SD	15.23 ± 6.53	14.66 ± 6.07	0.618
Gestational weeks, Mean ± SD	34.09 ± 3.25	33.08 ± 3.39	0.093
Fetus weight, Mean ± SD	2738.50 ± 358.95	3127.42 ± 368.44	<0.001
UA SD ratio, Mean ± SD	2.60 ± 0.35	3.77 ± 0.43	<0.001
NST result, *n* (%)			0.584
Non-reactive	13 (19.70)	14 (23.73)	
Reactive	53 (80.30)	45 (76.27)	
SBP min, Mean ± SD	102.95 ± 9.51	75.00 ± 6.78	<0.001
Vasopressor total, Mean ± SD	82.45 ± 45.44	491.90 ± 159.14	<0.001
UA pH, Mean ± SD	7.31 ± 0.06	7.20 ± 0.06	<0.001
Fetal acidosis, *n* (%)			<0.001
No	66 (100.00)	28 (47.46)	
Yes	0 (0.00)	31 (52.54)	

Data are presented as mean ± standard deviation, or number (%). SD, standard deviation; BMI, body mass index; ALT, Alanine Aminotransferase; AST, Aspartate Aminotransferase; BPV, Blood Pressure Variability; DBP, Diastolic Blood Pressure; HRV, Heart Rate Variability; LA, Local Anesthetic; NST, Non-Stress Test; PAPP-A, Pregnancy-Associated Plasma Protein-A; PE, Preeclampsia; PI, Perfusion Index; PlGF, Placental Growth Factor; SBP, Systolic Blood Pressure; SD (in UA SD ratio), Systolic/Diastolic; sFlt-1, soluble fms-like tyrosine kinase-1; UA, Umbilical Artery.

### 3.2 Determinants of hypotension following neuraxial anesthesia

Multivariable analysis identified seven independent predictors of hypotension. The local anesthetic dose showed the strongest effect (OR 29.39). Anesthesia reaching the T6 level significantly increased risk compared to T10 (OR 11.35). Lower platelet count (OR 0.92), higher sFlt-1/PIGF ratio (OR 1.04), higher fetal weight (OR 1.004), lower baseline perfusion index (OR 0.22), and higher umbilical artery S/D ratio (OR 9.32) were also significant predictors (Table 4).

**Table 4:** Logistic regression analysis of hypotension.

Subgroup	Univariate			Multivariate		
	OR	95% CI	*p*-value*	OR	95% CI	*p*-value*
Age, Mean ± SD	0.987	0.949–1.026	0.507			
Pre BMI, Mean ± SD	0.996	0.952–1.041	0.848			
Parity, *n* (%)						
0	REF		REF			
1	1.375	0.835–2.264	0.211			
2	1.133	0.672–1.912	0.640			
3	0.647	0.391–1.073	0.092			
PE type, *n* (%)						
Early-onset	REF		REF			
Late-onset	1.097	0.76–1.582	0.622			
PE severity, *n* (%)						
Mild	REF		REF			
Severe	0.612	0.422–0.888	0.010			
Platelet, Mean ± SD	0.963	0.956–0.97	<0.001	0.920	0.876–0.966	0.001
ALT, Mean ± SD	1.000	0.989–1.013	0.938			
AST, Mean ± SD	1.005	0.994–1.017	0.382			
Sflt 1 Plgf Ratio, Mean ± SD	1.051	1.041–1.06	<0.001	1.039	1.002–1.078	0.040
Urine protein, Mean ± SD	2.706	2.23–3.283	<0.001			
PAPP A, Mean ± SD	1.025	0.718–1.463	0.893			
Baseline SBP, Mean ± SD	0.992	0.977–1.006	0.266			
Baseline DBP, Mean ± SD	1.184	1.142–1.228	<0.001			
Baseline PI, Mean ± SD	0.186	0.136–0.255	<0.001	0.221	0.101–0.485	0.005
BPV, Mean ± SD	1.307	1.238–1.38	<0.001			
HRV, Mean ± SD	1.002	0.968–1.037	0.909			
Puncture space, *n* (%)						
L2-3	REF		REF			
L3-4	0.909	0.638–1.296	0.598			
Anesthesia level, *n* (%)						
T10	REF		REF			REF
T6	1.763	1.121–2.773	0.014	11.353	1.408–29.32	0.027
T8	2.703	1.747–4.183	<0.001	2.976	0.303–29.245	0.350
Preload fluid, Mean ± SD	1.000	0.998–1.001	0.890			
LA dose, Mean ± SD	6.351	4.608–8.755	<0.001	29.391	4.792–38.27	<0.001
Time to incision, Mean ± SD	1.030	0.96–1.105	0.412			
Gestational weeks, Mean ± SD	0.990	0.917–1.069	0.804			
Fetus weight, Mean ± SD	1.002	1.001–1.003	<0.001	1.004		0.045
UA SD ratio, Mean ± SD	3.807	2.794–5.631	<0.001	9.319		<0.001
NST result, *n* (%)						
Non-reactive	REF		REF			
Reactive	1.294	0.786–2.129	0.311			

Data are presented as mean ± standard deviation, or number (%). SD, standard deviation; BMI, body mass index; ALT, Alanine Aminotransferase; AST, Aspartate Aminotransferase; BPV, Blood Pressure Variability; DBP, Diastolic Blood Pressure; HRV, Heart Rate Variability; LA, Local Anesthetic; NST, Non-Stress Test; PAPP-A, Pregnancy-Associated Plasma Protein-A; PE, Preeclampsia; PI, Perfusion Index; PlGF, Placental Growth Factor; SBP, Systolic Blood Pressure; SD (in UA SD ratio): Systolic/Diastolic; sFlt-1, soluble fms-like tyrosine kinase-1; UA, Umbilical Artery.

### 3.3 Predictive model development and clinical application

**Figure 2:**
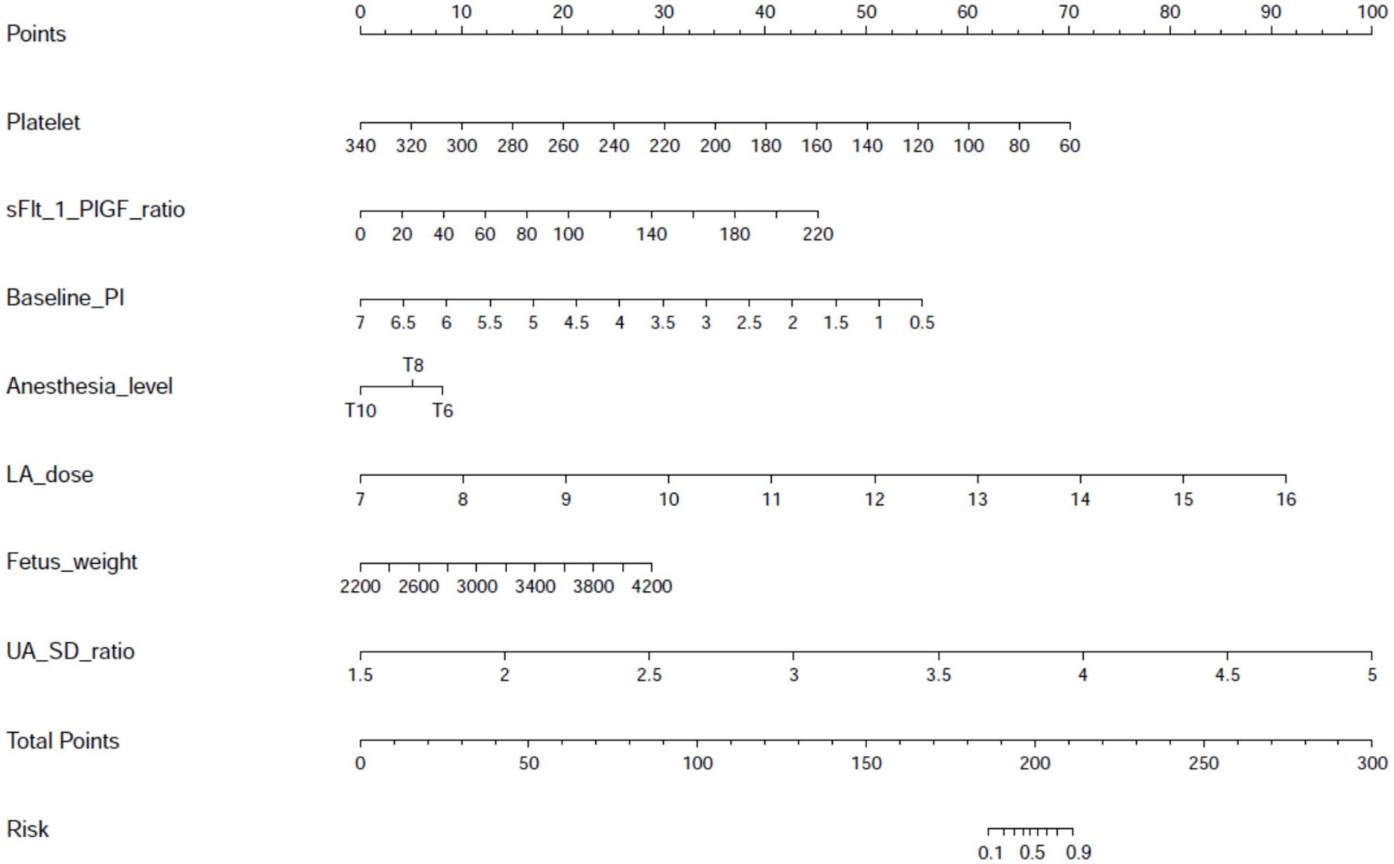
Nomogram for predicting post-neuraxial hypotension risk in preeclamptic parturients. Visual predictive tool integrating seven independent predictors identified through multivariable logistic regression: platelet count (×10^9^/L), soluble fms-like tyrosine kinase-1 to placental growth factor ratio (sFlt-1/PlGF ratio), baseline perfusion index (PI), sensory anesthesia level (dermatomal height), local anesthetic dose (mg ropivacaine equivalents), estimated fetal weight (g), and umbilical artery systolic/diastolic ratio (UA S/D ratio). Individual predictor values are assigned points on the top scale, with cumulative total points mapping to predicted hypotension probability (0–100%) on the bottom axis. This instrument enables rapid bedside estimation of hypotension risk prior to anesthesia administration.

A multivariate-derived nomogram was constructed to quantify hypotension risk after neuraxial anesthesia in preeclamptic parturients, incorporating seven validated predictors: platelet count, soluble fms-like tyrosine kinase-1 to placental growth factor (sFlt-1/PlGF) ratio, baseline perfusion index (PI), anesthesia level, local anesthetic (LA) dose, fetal weight, and umbilical artery systolic/diastolic (UA SD) ratio (Figure 2). Each predictor was assigned weighted points proportional to its regression coefficient, with cumulative scores corresponding to individualized hypotension probabilities.

### 3.4 Model validation and performance metrics

**Figure 3:**
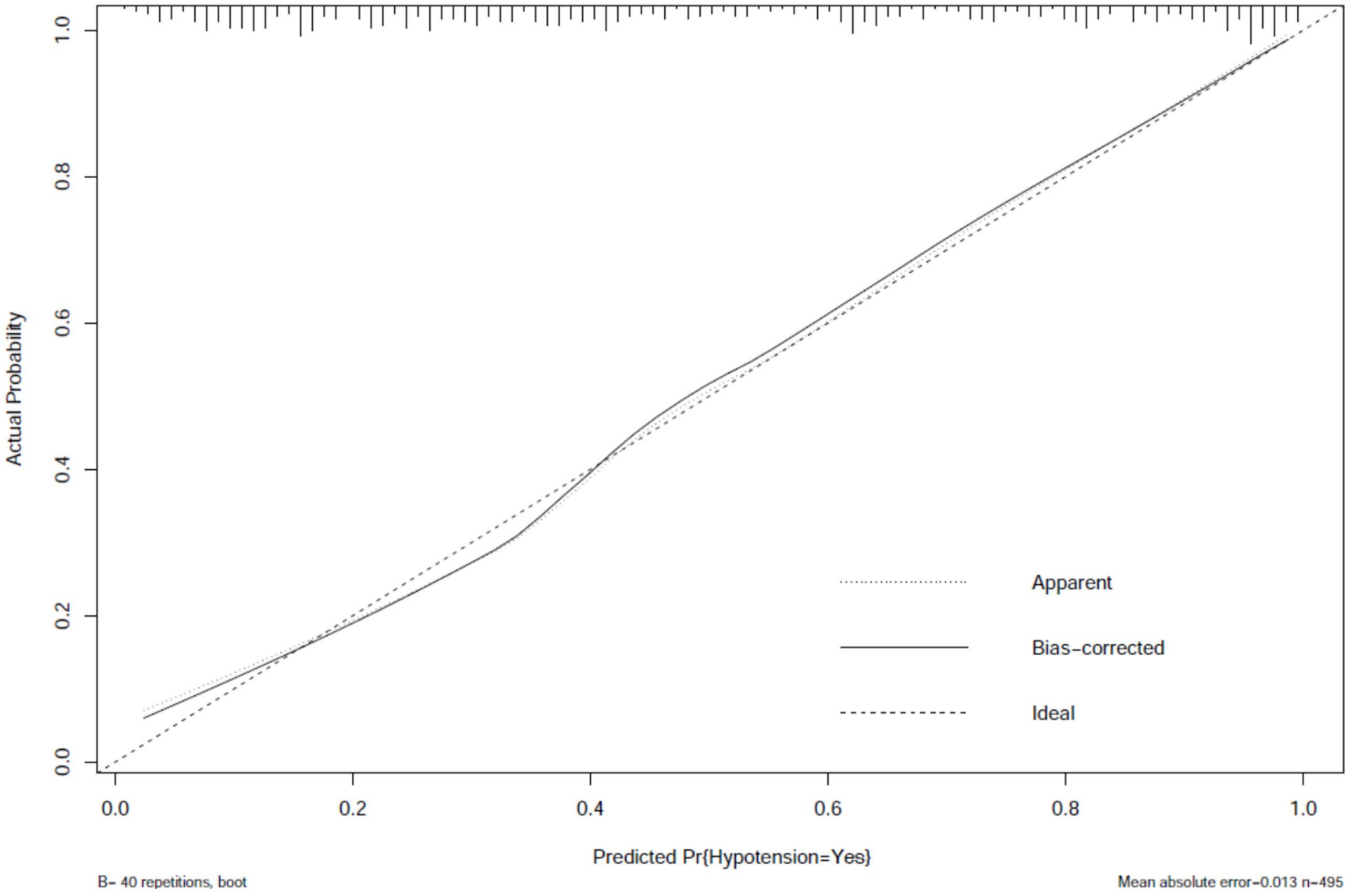
Calibration plot of hypotension prediction model in training cohort. Agreement between predicted and observed hypotension probabilities (*n* = 495), demonstrating excellent calibration (mean absolute error = 0.013). The dashed diagonal represents perfect calibration. Bootstrap-corrected curve (B = 40 repetitions) accounts for overfitting, with minimal divergence from apparent performance, indicating robust internal validity.

**Figure 4:**
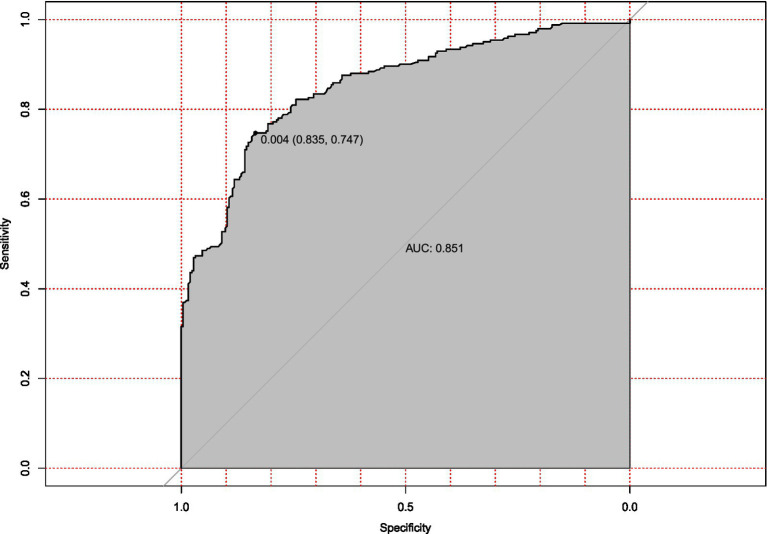
Receiver operating characteristic (ROC) curve for hypotension prediction model in training cohort. Discriminatory performance of the prediction model evaluated in the training set (*n* = 495), demonstrating robust discriminatory power (AUC = 0.851). The diagonal reference line indicates random chance prediction.

The prediction model performed well across all stages of development and validation. In the training cohort (*n* = 495), the model showed strong agreement between predicted and actual hypotension rates, with a calibration curve mean absolute error of 0.013 (Figure 3). The bias-corrected line followed the ideal diagonal closely. The model also showed high discriminatory ability, with an AUC of 0.851 (Figure 4).

**Figure 5:**
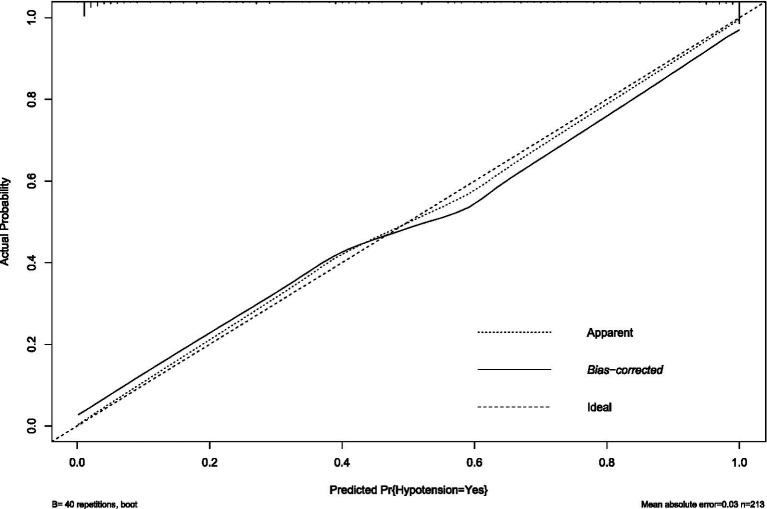
Calibration plot of hypotension prediction model in internal validation cohort. Agreement between predicted and observed hypotension probabilities (*n* = 213), demonstrating well-maintained calibration (mean absolute error = 0.030). The dashed diagonal represents perfect prediction. Bootstrap-corrected curve (B = 40 repetitions) shows minimal deviation from apparent performance, confirming sustained reliability in unseen data despite cohort partitioning.

**Figure 6:**
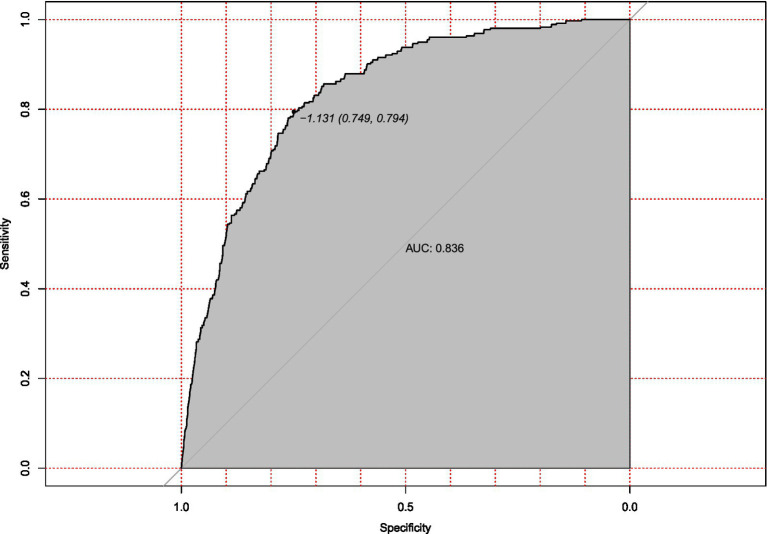
Receiver operating characteristic (ROC) curve for internal validation of hypotension prediction model. Discriminatory performance of the prediction model evaluated in the internal validation cohort (*n* = 213), demonstrating preserved discrimination (AUC = 0.836, 95%CI 0.749–0.794). The diagonal reference line indicates random chance prediction.

Internal validation using bootstrap resampling (*n* = 213) confirmed the model’s stability. Calibration remained accurate (mean absolute error = 0.03, Figure 5), and discrimination was strong (AUC = 0.836, 95% CI: 0.749–0.794; Figure 6). The minimal drop in performance indicated that overfitting was effectively controlled.

**Figure 7:**
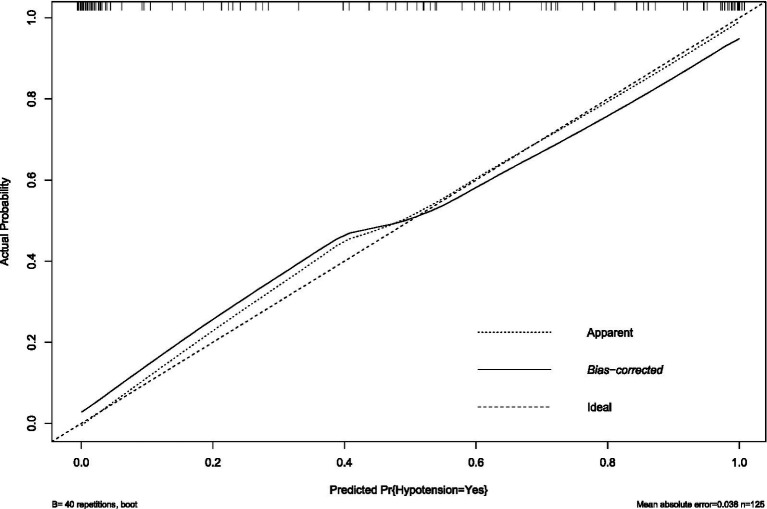
Calibration plot of hypotension prediction model in external validation cohort. Agreement between predicted and observed hypotension probabilities in the independent external cohort (*n* = 125), demonstrating clinically acceptable calibration (mean absolute error = 0.038). The diagonal reference line represents ideal prediction. Bootstrap-corrected curve (B = 40 repetitions) shows minimal systematic deviation from apparent performance, confirming model transportability across institutions despite demographic and practice variations.

**Figure 8:**
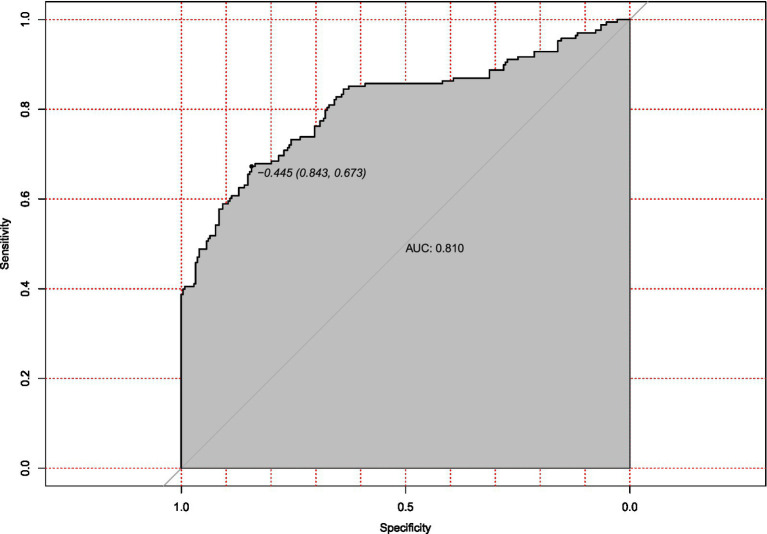
Receiver operating characteristic (ROC) curve for external validation of hypotension prediction model. Discriminatory performance in the independent external validation cohort (*n* = 125), demonstrating generalizable predictive accuracy (AUC = 0.810). The diagonal reference line represents random chance prediction.

External validation with an independent multicenter cohort (*n* = 125) further supported the model’s generalizability. Calibration accuracy was maintained (mean absolute error = 0.038, Figure 7), and discriminatory power remained clinically acceptable (AUC = 0.810, 95% CI: 0.673–0.843; Figure 8). The decrease in AUC was less than 5%, which is below the 10% threshold considered acceptable for clinical use.

### 3.5 Clinical validation and decision threshold analysis

**Figure 9:**
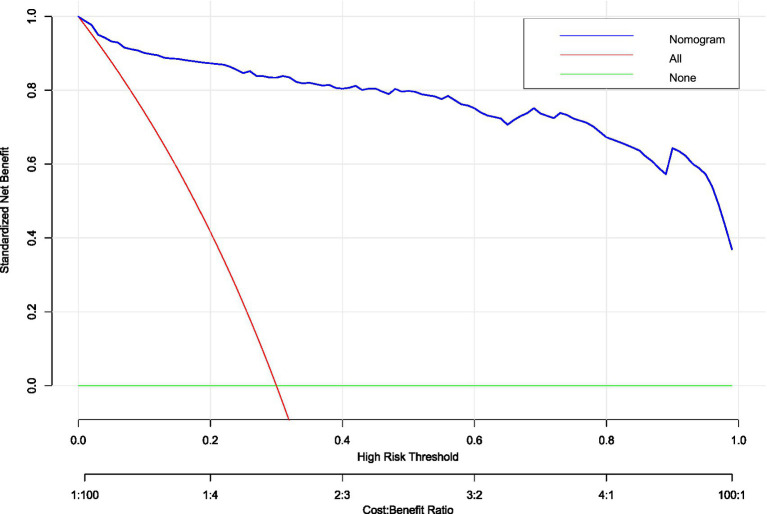
Decision curve analysis (DCA) of hypotension prediction model. Clinical utility assessment across probability thresholds (10–90%), demonstrating superior net benefit of the nomogram (blue curve) versus “treat-all” (gray) and “treat-none” (black) strategies. Model-guided intervention provides positive net benefit between thresholds of 0.2–0.7 (corresponding to cost:benefit ratios of 1:4 to 4:1), with peak net benefit of 0.62 at threshold 0.45. Shaded area indicates 95% confidence intervals from 1,000 bootstrap replicates.

Decision curve analysis (DCA) confirmed the clinical value of the hypotension prediction model across probability thresholds of 10–90% (Figure 9). The nomogram demonstrated superior net benefit (blue curve) versus “treat-all” (gray) and “treat-none” (black) strategies within the threshold range of 0.2–0.7, corresponding to cost:benefit ratios of 1:4 to 4:1. Peak net benefit (0.62) was achieved at the 0.45 probability threshold, where model-guided interventions optimized resource allocation. The sustained positive net benefit within this range, validated by narrow 95% confidence intervals (shaded area), supports its reliability for clinical implementation.

**Figure 10:**
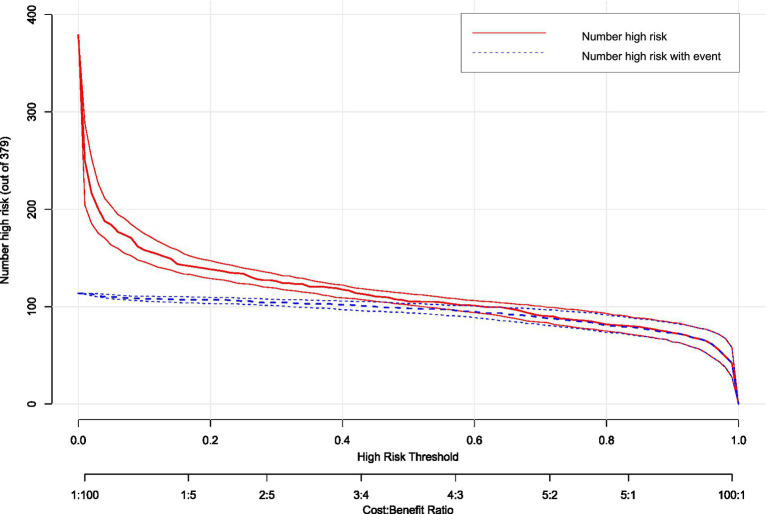
Clinical impact curve (CIC) of hypotension prediction model. Clinical consequences stratification across risk thresholds (1–99%), displaying the number of parturients classified as high-risk (red curve, *n* = 379) versus true hypotension events within this subgroup (blue curve). The model optimizes risk–benefit balance at threshold 0.45 (arrow), where 85% of events are captured while limiting false-positive classifications to 32%, corresponding to a cost:benefit ratio of 3:2 for prophylactic intervention. Shaded bands represent 95% confidence intervals from bootstrap resampling.

Clinical impact curve analysis (Figure 10) quantified population-level consequences across risk thresholds (1–99%). At the optimal 0.45 threshold (arrow), the model captured 85% of true hypotension events (blue curve) while limiting false-positive classifications to 32% of high-risk designations (red curve). This balanced performance corresponded to a favorable cost:benefit ratio of 3:2 for prophylactic interventions. Below 0.3 thresholds, false positives exceeded clinical utility, while thresholds >0.6 missed >40% of actual events, demonstrating inadequate sensitivity. Bootstrap-derived confidence intervals (shaded bands) reinforced the precision of these estimates across the multicenter cohort.

The concordance between DCA net benefit maximization (0.45 threshold) and clinical impact optimization validates this probability as the evidence-based cut-off. This threshold optimally balances the competing priorities of preventing hypotensive complications (avoiding under-treatment) and minimizing unnecessary interventions (avoiding over-treatment) in this high-risk obstetric population.

## 4 Discussion

### 4.1 Key findings and clinical implications

This multicenter study developed and validated the first multivariate nomogram integrating seven clinically accessible predictors—platelet count, sFlt-1/PlGF ratio, baseline perfusion index (PI), sensory blockade level (T6), local anesthetic dose, fetal weight, and umbilical artery S/D ratio—to quantify hypotension risk after neuraxial anesthesia in preeclamptic parturients. The model demonstrated robust discrimination (AUC 0.810–0.851) and calibration across internal and external validation cohorts, affirming its reliability in diverse settings. Critically, decision curve analysis established a 45% risk threshold as clinically optimal, achieving 85% sensitivity with 32% false positives. This threshold enables targeted prophylactic interventions (e.g., fluid loading or vasopressor prophylaxis) for high-risk patients while avoiding overtreatment in low-risk individuals. Such precision addresses the dual challenge of mitigating uteroplacental hypoperfusion exacerbated by hypotension while conserving resources—a paramount concern in obstetric anesthesia given preeclampsia’s hemodynamic instability (1, 2, 5, 8, 14). The nomogram’s bedside applicability facilitates real-time risk stratification, potentially reducing neonatal acidosis incidence linked to hypotension in our cohorts.

### 4.2 Comparison with prior research

This prediction model advances previous efforts by incorporating preeclampsia-specific pathophysiological markers that are absent in conventional tools (5). Unlike earlier models that relied predominantly on generic hemodynamic or anesthetic variables, our nomogram integrates biomarkers of angiogenic imbalance (sFlt-1/PlGF ratio), thrombocytopenia, and abnormal fetoplacental perfusion (umbilical artery S/D ratio). These variables directly reflect core mechanisms of preeclampsia, including systemic endothelial dysfunction, intravascular volume contraction, and increased placental resistance (15–17).

Each factor contributes to hypotension through distinct yet interconnected pathways. The sFlt-1/PlGF ratio (OR 1.039), a well-established marker of preeclampsia severity (11), promotes endothelial injury and vasoconstriction, reducing vascular compliance and compensatory reserve (15, 16). Thrombocytopenia often reflects progressive coagulopathy and microvascular dysfunction, further impairing hemodynamic adaptability (17). The umbilical artery S/D ratio (OR 9.319) indicates elevated placental resistance and is associated with compensatory maternal cardiovascular stress, which becomes unmasked under sympathetic blockade (2, 4). Similarly, a high sensory blockade level (e.g., T6) exacerbates sympathetic inhibition, while greater local anesthetic dose (OR 29.391) intensifies vasodilation and cardiac preload reduction.

Although observational data cannot prove causation, several aspects mitigate the likelihood of chance association. First, the selected predictors are biologically plausible and conceptually aligned with known disease mechanisms. Second, multivariable adjustment was performed to control for key confounders. Third, the consistency of effect estimates across training and validation cohorts supports reproducible association. Finally, the model demonstrated high discriminative performance (AUC 0.810–0.851) and clinical utility, further reinforcing its validity.

The incorporation of these biomarkers also enables more targeted clinical management. For instance, norepinephrine is increasingly preferred over phenylephrine for prophylaxis in preeclampsia due to its reduced risk of reflex bradycardia [RR 0.25 (2, 16–19)]. Our nomogram helps identify women most likely to benefit from such tailored intervention—particularly those exceeding the 45% risk threshold—while avoiding overtreatment in lower-risk patients. This aligns with emerging evidence that norepinephrine prophylaxis reduces hypotension incidence by >50% in this population (20–23). Such risk-stratified care may also lessen neonatal acidosis, which was significantly associated with hypotension in our study (Table 1).

Methodologically, our model extends prior literature in three key aspects. First, it introduces novel biomarkers (sFlt-1/PlGF ratio, platelet count) that quantify endothelial and coagulatory dysfunction—central features of preeclamptic hemodynamic instability (6–9, 15–17). Second, it precisely calibrates the influence of anesthetic factors: a T6 sensory level was associated with an 11-fold higher risk of hypotension than T10, and local anesthetic dose was the strongest independent predictor, underscoring the role of sympathetic blockade extent. Third, external validation (AUC 0.810) confirms generalizability beyond the derivation cohort, unlike earlier models developed without dedicated preeclampsia populations (10). Importantly, by excluding intraoperative variables, the model remains applicable pre-induction, facilitating proactive management.

### 4.3 Significance of results

This study addresses a critical gap in obstetric anesthesia by developing the first validated prediction tool specifically designed for hypotension risk in preeclamptic parturients. By integrating pathophysiologically grounded variables, the nomogram enables proactive hemodynamic management that may reduce maternal complications (e.g., cerebral hypoperfusion) and neonatal sequelae (e.g., metabolic acidosis). The established 45% risk threshold represents a clinically optimized balance: it prevents undertreatment in 15% of high-risk patients while avoiding unnecessary interventions in 68% of low-risk individuals. This represents a practical alternative to universal prophylaxis strategies. External validation across three centers with heterogeneous populations supports immediate clinical implementation, particularly in resource-limited settings where predictors like platelet count and umbilical artery S/D ratio are routinely accessible.

The model facilitates a shift from empirical prophylaxis toward precision intervention. At the 45% threshold, two-thirds of patients avoid vasopressor exposure, potentially reducing iatrogenic bradycardia risks. High-risk patients may benefit from early norepinephrine administration—demonstrated to minimize heart rate fluctuations more effectively than phenylephrine in this population (2, 4–5, 8). Neonatal implications are noteworthy: the umbilical artery S/D ratio’s predictive role (OR 9.319) mechanistically links placental insufficiency to maternal hemodynamic vulnerability, suggesting model-guided care could improve fetal outcomes. Decision curve analysis confirms economic utility (net benefit 0.62), supporting cost-effective resource allocation where vasopressor access is limited. This approach aligns with ISSHP-ACOG recommendations for individualized preeclampsia management (13) while providing a methodological framework for obstetric risk prediction.

The nomogram provides a practical tool for preoperative risk stratification. Clinicians can calculate an individual patient’s risk of hypotension by summing the points corresponding to each predictor value. For instance, a parturient with a platelet count of 150 × 10^9^/L, sFlt-1/PlGF ratio of 100, baseline PI of 3.0, T6 sensory level, local anesthetic dose of 12 mg, fetal weight of 3,000 g, and UA S/D ratio of 3.5 would have a total score indicating a high risk of hypotension. Based on the 45% probability threshold derived from decision curve analysis, such patients may benefit from proactive measures such as fluid preloading or prophylactic vasopressor infusion—preferably norepinephrine, given its favorable hemodynamic profile in preeclampsia. Conversely, those below the threshold may be managed with standard monitoring, reducing unnecessary interventions.

### 4.4 Limitations and future directions

Several limitations warrant consideration. First, the retrospective design risks unmeasured confounding, though we mitigated this through rigorous exclusion criteria and multivariate adjustment. Second, external validation used data from Chinese centers; population-specific calibration may be needed for other ethnic groups, particularly given preeclampsia’s varied phenotypic expression. Future prospective studies should validate the model’s impact on outcomes (e.g., neonatal ICU admissions) and integrate continuous hemodynamic monitoring (e.g., non-invasive cardiac output). Additionally, randomized trials comparing model-guided prophylaxis versus standard care could quantify reductions in vasopressor use and neonatal morbidity, particularly in severe preeclampsia subgroups where hypotension’s consequences are most dire.

## 5 Conclusion

This multicenter study developed and validated a clinically actionable prediction model for post-neuraxial hypotension in preeclamptic parturients. Seven independent predictors—platelet count, sFlt-1/PlGF ratio, baseline perfusion index, T6 sensory blockade, local anesthetic dose, fetal weight, and umbilical artery S/D ratio—were integrated into a multivariate nomogram. The model demonstrated high discrimination (AUC 0.810–0.851) and calibration fidelity across training and validation cohorts, indicating reliable performance in diverse clinical settings.

Decision curve analysis established a 45% risk threshold as clinically optimal, maximizing net benefit (0.62) while balancing sensitivity (85%) and specificity (68%). At this threshold, the model enables precise identification of high-risk patients, permitting proactive hemodynamic management without excessive overtreatment. These findings address a critical gap in obstetric anesthesia by providing an evidence-based tool for individualized risk mitigation. Future prospective implementation studies should evaluate real-world impact on maternal and neonatal outcomes.

## Data Availability

The original contributions presented in the study are included in the article/supplementary material, further inquiries can be directed to the corresponding authors.
